# Vulvar ulcerative cutaneous tuberculosis secondary to genital tract tuberculosis^☆^

**DOI:** 10.1016/j.abd.2021.11.004

**Published:** 2022-09-16

**Authors:** Jiangfan Yu, Wenxia Huang, Guiying Zhang, Rong Xiao

**Affiliations:** a Department of Dermatology, Second Xiangya Hospital of Central South University, Changsha, China; b Department of Dermatology, The Fifth People Hospital of Hainan Province, China

**Keywords:** Cutaneous, Female genital, Skin ulcer, Tuberculosis

## Abstract

Tuberculosis is a chronic infectious disease that gradually poses a certain threat to public health and economic growth. Tuberculosis typically affects the lungs, pleura, and lymph nodes and rarely the skin. Cutaneous tuberculosis manifesting as ulcerated lesions is also rare and often misdiagnosed and missed by clinicians. Here, the authors report a 29-year-old female patient presenting a vulvar ulcer for 10 months, accompanied by irregular menstruation and increased vaginal secretions. After a skin biopsy and endometrial PCR testing, it was finally diagnosed as vulvar ulcerative cutaneous tuberculosis secondary to genital tuberculosis. Anti-tuberculosis treatment was effective. Cutaneous tuberculosis is called a great imitator. In order to facilitate the diagnosis and treatment of tuberculosis by clinicians, the authors systematically reviewed this disease as well.

Tuberculosis (TB) is a public health concern caused by infection with *Mycobacterium tuberculosis*, typically affecting the lungs (80%) and less than 1% is cutaneous TB (CTB).^1^ The clinical appearance of CTB is quite varied, the most predominant form is lupus vulgaris, while ulcerative lesions are rare, constituting 2% of CTB cases.^2^ CTB is often misdiagnosed and missed in daily work. In this report, the authors introduce a rare case of vulvar ulcerative CTB secondary to genital TB and review the relevant literature. A 29-year-old female patient presented with a vulvar ulcer for 10 months. Since the second child was delivered by cesarean section, her menstruation has become irregular, often accompanied by bloody leucorrhea, and finally, secondary amenorrhea appeared. No fever, night sweats, and weight loss. Misdiagnosed as a common bacterial infection, Behcet's disease, or pyoderma gangrenosum in local clinics, but anti-infective treatment and glucocorticoid therapy were not effective. Physical examination found an ulcer with purulent exudation and infiltrative borders in the vulva (Fig. 1a). The ratio of leukocyte and neutrophil was increased. Purified protein derivative skin test and tuberculin test were positive. Hysteroscopy revealed bilateral adhesion of uterine horns and double fallopian tubes. Fluorescent quantitative PCR detection of *Mycobacterium tuberculosis* in endometrial specimens was positive. Histopathology of the vulvar ulcer revealed granulomas with caseous necrosis and lymphocytic infiltrate, which confirmed the diagnosis of vulvar ulcerative CTB secondary to genital TB. Because the patient’s uric acid was slightly elevated, pyrazinamide may cause an increase in uric acid and the patient had some gastrointestinal reactions when taking rifampicin, isoniazid, and ethambutol, the authors did not use pyrazinamide under a comprehensive consideration. The ulcer had healed by the third month and was followed up for five years without recurrence (Fig. 1b). CTB is generally a secondary infection that occurs in patients with internal organ TB.^2^, ^3^ The general clinical manifestation of ulcerative CTB is an ulcerative lesion with irregular edges and clear boundaries with a purulent base.^4^ The presence of yellow satellite nodules around the ulcer is characteristic of ulcerative CTB.^5^ In addition to the vulvar lesion (Fig. 2), endometrial PCR confirmed endometrial TB in our case. It is a common type of genital TB, which usually manifested as abnormal secretions, decreased menstruation, or secondary amenorrhea,^6^ and the patient the authors reported has similar clinical manifestations above. Genital TB generally affects the fallopian tubes almost 100% and the vulva rarely 0.07%.^7^ Here, ulcerative CTB of the vulva is an external cue sign of genital TB. Treatment options for various types of CTB are the same as TB, and last for at least 6 months.^8^, ^9^ CTB will not heal itself, and it may be life-threatening if the improper or untimely treatment causes widespread transmission of *Mycobacterium tuberculosis*.^10^ Therefore, the early diagnosis and treatment of CTB are particularly important.

**Figure 1:**
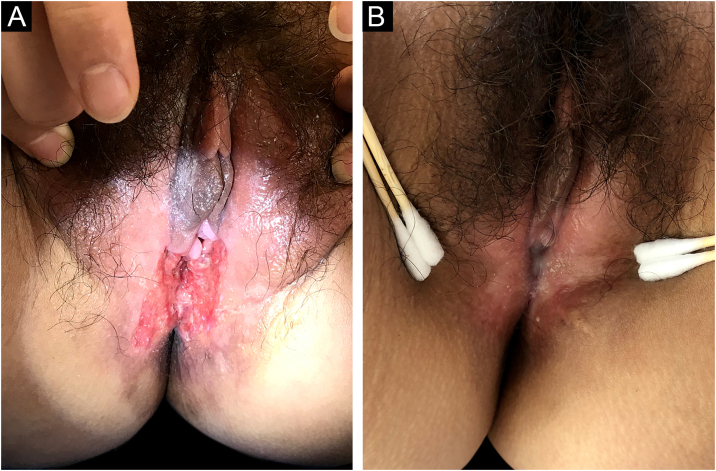
Clinical images. (a) The appearance of the ulcer before treatment. (b) The ulcer healed after treatment.

**Figure 2:**
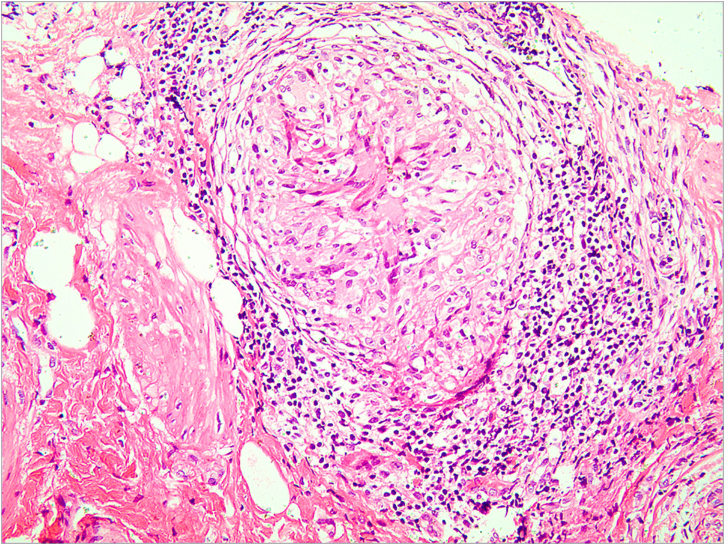
Hematoxylin & eosin, ×400. A granuloma with caseous necrosis and lymphocytic infiltration.

## Financial support

None declared.

## Authors’ contributions

Jiangfan Yu: Contributed to the study concept and design; wrote the manuscript.

Wenxia Huang: Conducted data collection and statistical analysis.

Guiying Zhang: Effectively participated in the research guidance, the propaedeutic and therapeutic conduct of the studied cases.

Rong Xiao: Contributed to the study concept and design; made a critical review of the literature and finally approved the final version of the manuscript.

## Conflict of interest

None declared.
